# Temperature and population density influence SARS-CoV-2 transmission in the absence of nonpharmaceutical interventions

**DOI:** 10.1073/pnas.2019284118

**Published:** 2021-06-08

**Authors:** Thomas P. Smith, Seth Flaxman, Amanda S. Gallinat, Sylvia P. Kinosian, Michael Stemkovski, H. Juliette T. Unwin, Oliver J. Watson, Charles Whittaker, Lorenzo Cattarino, Ilaria Dorigatti, Michael Tristem, William D. Pearse

**Affiliations:** ^a^Department of Life Sciences, Imperial College London, Ascot SL5 7PY, United Kingdom;; ^b^Department of Mathematics, Imperial College London, London SW7 2AZ, United Kingdom;; ^c^Department of Biology, Utah State University, Logan, UT 84322;; ^d^Ecology Center, Utah State University, Logan, UT 84322;; ^e^MRC Centre for Global Infectious Disease Analysis, Imperial College London, London W2 1PG, United Kingdom

**Keywords:** SARS-CoV-2, transmission, climate, seasonality, epidemiology

## Abstract

As COVID-19 continues to spread across the world, it is increasingly important to understand the factors that influence its transmission. Seasonal variation driven by responses to changing environment has been shown to affect the transmission intensity of several coronaviruses. However, the impact of the environment on severe acute respiratory syndrome coronavirus 2 (SARS-CoV-2) remains largely unknown, and thus seasonal variation remains a source of uncertainty in forecasts of SARS-CoV-2 transmission. Here we address this issue by assessing the association of temperature, humidity, ultraviolet radiation, and population density with estimates of transmission rate (*R*). Using data from the United States, we explore correlates of transmission across US states using comparative regression and integrative epidemiological modeling. We find that policy intervention (“lockdown”) and reductions in individuals’ mobility are the major predictors of SARS-CoV-2 transmission rates, but, in their absence, lower temperatures and higher population densities are correlated with increased SARS-CoV-2 transmission. Our results show that summer weather cannot be considered a substitute for mitigation policies, but that lower autumn and winter temperatures may lead to an increase in transmission intensity in the absence of policy interventions or behavioral changes. We outline how this information may improve the forecasting of COVID-19, reveal its future seasonal dynamics, and inform intervention policies.

## Significance

There is still much to be understood about the factors influencing the ecology and epidemiology of COVID-19. In particular, whether environmental variation is likely to drive seasonal changes in SARS-CoV-2 transmission dynamics is largely unknown. We investigate the effects of the environment on SARS-CoV-2 transmission rates across the United States and then incorporate the most important environmental parameters into an epidemiological model. We show that temperature and population density can be important factors in transmission but only in the absence of mobility-restricting policy measures, although particularly strong policy measures may be required to mitigate the highest population densities. Our findings improve our understanding of the drivers of COVID-19 transmission and highlight areas in which policy decisions can be proactive.

In late 2019, a novel coronavirus originating in Wuhan City (Hubei, China) (1) began to rapidly spread through the human population. Since March 2020, this disease, COVID-19, has been recognized as a global pandemic by the World Health Organization. The causative agent, severe acute respiratory syndrome coronavirus 2 (SARS-CoV-2), is a close relative of the 2003 SARS coronavirus (1), although it appears to have several differences, including a higher basic reproduction number ($R_{0}$; the average number of people infected by a carrier at the onset of an epidemic) (2). Understanding the factors influencing SARS-CoV-2 transmission is key for understanding the current patterns of transmission and for refining predictions of the future spread of SARS-CoV-2. Other coronaviruses display seasonal cycles of transmission, and up to 30% of seasonal “colds” are caused by coronaviruses (3). Thus, as many countries alter and relax the nonpharmaceutical interventions initially imposed to control COVID-19, there is a pressing need to understand whether environmental factors will enhance or drive additional “waves” of COVID-19 outbreaks as places move through seasonal climate patterns (4).

SARS-CoV-2 is an enveloped RNA virus which is structurally (if not phylogenetically) similar to other RNA viruses such as influenza, Middle East respiratory syndrome, and HcoV-NL63 (5) that are known to display seasonal dynamics due to their physical properties. For example, high temperatures and low humidity can have a negative effect on influenza transmission by reducing the efficiency of respiratory droplet transmission (6, 7). Similar effects are seen in transmission of coronaviruses (8–10), where high environmental temperatures break down viral lipid layers to inactivate virus particles that are in the air or deposited on surfaces (9, 11). However, assessing the role of environment during a disease outbreak is challenging (12) because human factors such as population density, herd immunity, and behavior are likely the main drivers of transmission (13–16). Moreover, the nonpharmaceutical control measures and behavioral changes in response to COVID-19 have been unprecedented in the modern era. These difficulties have hindered the quantification of the impact of environment on SARS-CoV-2 transmission, making it harder to generalize and synthesize observations across regions with their differing climates. Despite these caveats, various early studies have already reported effects of environmental variables such as temperature, humidity, ultraviolet (UV) levels, and wind speed on the transmission of SARS-CoV-2 (16–24). While, in general, most studies appear to support increased transmission rates under cool, dry conditions (18), conflicting results have been observed (21, 25), and, collectively, the environmental signal appears to be weak (4). Much of the variability in these early results is likely due to the use of inappropriate response variables (such as cases or fatalities) which fail to capture the intrinsic variations in transmission intensity driven by the effects of nonpharmaceutical intervention measures (4). Furthermore, COVID-19 has taken hold in many places with diverse climates, and there are obvious examples of high transmission rates under warmer conditions, such as in Brazil (26), India (27), and Iran (28).

Accurate assessment of the role environmental factors have played so far in the spread of SARS-CoV-2 may provide insight into the future seasonality of the disease. This is because seasonal outbreaks of viruses are often driven by their responses to favorable (seasonal) changes in weather (29). Most epidemiological forecasts make use of some variant of the Susceptible–Infected–Recovered (SIR) framework and/or focus on the impacts of government-level mitigation (e.g., refs. 30 and 31). Few epidemiological models incorporate environmental impacts, and, when they do, they assume COVID-19 responds in a manner identical to related coronaviruses, because we lack data on SARS-CoV-2’s environmental (and thus seasonal) responses (e.g., ref. 18). This is despite theoretical demonstrations of the potential role of environment in driving future seasonality of SARS-CoV-2 (22, 32) and the empirical evidence in structurally similar viruses outlined above. Efforts to incorporate climate into COVID-19 forecasting have focused on regression-type models of cases and fatalities (e.g., ref. 17), which are unreliable when diseases are in the growth/expansion phase (33). Furthermore, such models conflate environmental controls on occurrence with other drivers such as public health interventions (e.g., the effects of lockdown measures to contain the pandemic) (33), as both are changing similarly through time. Such models are unlikely to yield useful insights and may be misleading to policy makers (12). To address this knowledge gap, there is a need for a true synthesis of environmental modeling with well-established epidemiological approaches.

Here we investigate the role of environment in the transmission of SARS-CoV-2 by incorporating environmental factors into an existing epidemiological framework that has been applied globally (34–36), and to the United States in particular (37). The United States is a large country with great variation in climate across which case and policy intervention data are comparable, permitting us to disentangle the role of environmental drivers in SARS-CoV-2 transmission. We begin by exploring associations between the environment (temperature, humidity, UV radiation, and population density) and transmission intensity independently estimated before and during stay-at-home orders (henceforth termed “lockdown”). We used the basic reproduction number ($R_{0}$) for our prelockdown transmission intensity estimates, and the time-varying reproduction number ($R_{t}$, the reproduction number, R, at a given time, t) averaged across an appropriate time window for our during-lockdown estimates. Our independent analysis of $R_{0}$ focuses on a single snapshot (the beginning) of the virus’s outbreak in each state, and reveals whether differences in transmission across states are correlated with differences in environment across space at that snapshot in time. Critically, this independent analysis allows us to investigate the role of environment in the absence of any temporally correlated changes in climate and transmission rate. After confirming a potential role for the environment, we verify and more accurately quantify the relative roles of temperature and population density by integrating them into an existing semimechanistic epidemiological framework (37). While we find strong evidence that temperature and population density are associated with SARS-CoV-2 transmission, we emphasize that our findings also reconfirm that the major drivers of transmission rates are public policy and individual behavior. Through our use of existing, robust sources of forecasts and models, our findings can be easily incorporated into workflows already used by policy makers, as we detail here.

## Results

When analyzed jointly, the $R_{0}$ of all US states are fairly well predicted by all explanatory variables included in the regression model (i.e. population density, temperature, absolute humidity, and UV radiation), with an overall model $r^{2}$ of 60% (*SI Appendix*, Table S1). However, UV radiation is a very weak predictor of $R_{0}$, while temperature and absolute humidity show sufficiently strong correlations with each other (r=0.85) that we cannot disentangle their contributions to $R_{0}$ due to high inflation of variances (*SI Appendix*, Table S1). This is further demonstrated through principal components analysis, where temperature and absolute humidity strongly load onto the same principal component axis (*SI Appendix*, Fig. S1). We therefore focused on temperature as the best-fitting climate variable (assessed by Pearson’s r; *SI Appendix*, Table S2).

We regressed prelockdown $R_{0}$ and during-lockdown $R_{t}$ (defined as the mean $R_{t}$ recorded over the 14-d period following a stay-at-home order) estimates against temperature and $\log_{10}$-transformed population density, with lockdown as a binary interaction term. This model explicitly tests for not just the effect of temperature, population density, and lockdown on transmission but also whether the relationship between $R_{0}$ and temperature or population density differs under lockdown.

**Fig. 1.**
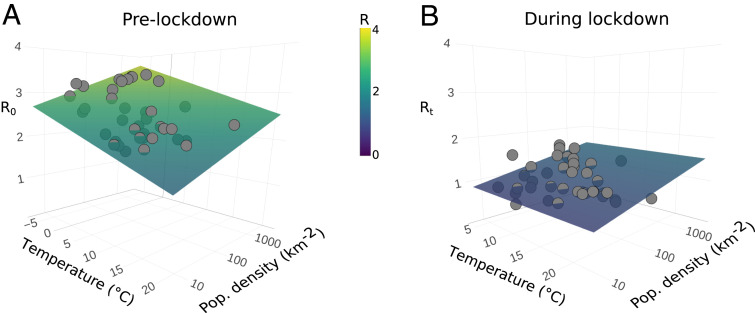
$R_{0}$ is affected by the environment, but the impact of lockdown is greater. (*A*) $R_{0}$ plotted against temperature (averaged across the 2 wk prior to the $R_{0}$ estimate) and $\log_{10}$-transformed population density (people per ${km}^{2}$) for each US state (gray points). Surface shows the predicted $R_{0}$ from the regression model (Table 1). Temperature has a negative effect on $R_{0}$ at state level in the United States, while population density has a positive effect (Table 1). (*B*) The mean $R_{t}$ for the 2 wk following a statewide stay-at-home mandate (i.e., during lockdown) plotted against average daily temperature for the same period and $\log_{10}$-transformed population density. The effects of temperature and population density are much weaker in the mobility-restricted data, and R is reduced overall. The same color scale, given in the center of the figure, is used across both subplots. A two-dimensional (2D) representation of these results is shown in *SI Appendix*, Fig. S2.

We find that, in the absence of lockdown, $R_{0}$ significantly increases with population density and decreases with temperature (Fig. 1*A*; both p<0.001; Table 1). However, lockdown significantly decreases R overall (Table 1 and Fig. 1*B*), and, moreover, there is a significant interaction between lockdown and temperature (p<0.001; Table 1), as, under the effects of lockdown, the temperature coefficient is essentially reduced to zero; that is, lockdown mitigates the effects of climate on transmission.

**Table 1.** Population (Pop) density and temperature are drivers of $R_{0}$ at state level in the United States, but the effect of lockdown is greater

	Coefficient	SE	*t* value	*p* value
(Intercept)	2.41	0.050	48.4	<0.001*
Temperature	−0.30	0.048	−6.13	<0.001*
Pop density	0.19	0.045	4.20	<0.001*
Lockdown	−1.29	0.072	−17.8	<0.001*
Temperature contrast	0.30	0.075	3.92	<0.001*
Pop density contrast	−0.07	0.064	−1.09	0.28

Here, $r^{2}=89\%$, $F_{5,74}=123$, and p<0.001. Coefficient estimates are when predictors are scaled to have mean = 0 and SD = 1. Scaling our explanatory variables means our coefficients are measures of the relative importance of each variable. Contrasts are changes to coefficients when lockdown is in place; that is, a positive temperature contrast means that the temperature coefficient increases by that value under lockdown conditions. Here, temperature is a greater driver of $R_{0}$ than population density (${log}_{10{}^{-}}$transformed), but only in the absence of nonpharmaceutical interventions (lockdown).

* Here, p<0.05.

**Fig. 2.**
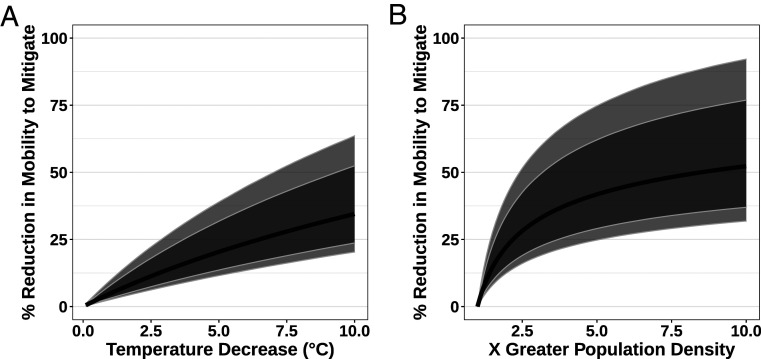
Average mobility reductions required to mitigate differences in temperature and population density. This figure shows the percent reduction in average mobility (measuring retail, recreation, grocery, pharmacy, and workplace trips) needed to compensate for (*A*) a given temperature- or (*B*) population density-driven increase in $R_{t}$. These calculations assume a “background” $R_{0}$ of one and a baseline “background” mobility [defined as “0” by Google (38)]. Solid lines represent the median mobility reduction required; dark gray and light gray envelopes represent the 75% and 90% posterior credibility intervals, respectively.

The strong correlates of population density and temperature on $R_{0}$ across the United States were echoed in our climate-driven Bayesian modeling of daily variation in $R_{t}$. Posterior medians of the scaled coefficients of ($\log_{10}$-transformed) population density and daily temperature were 0.68 and −0.48, respectively. These coefficients were strongly supported (both Bayesian probabilities of >99.9%), and suggest that greater population density is approximately 1.4 times greater a driver of higher transmission than colder temperature (0.68/0.48≈1.4). Changes in mobility (such as those induced by stay-at-home measures) have the potential to mitigate these impacts of both population density and temperature (Fig. 2). Our model suggests that even quite large variation in underlying transmission driven by variation either in temperature through time or in population density across space can be mitigated by reductions in mobility (see also *SI Appendix*, Fig. S6). Critically, however, the posterior distributions are skewed, particularly for population density: High population density may be difficult to mitigate except through large mobility reductions (as shown by the credibility intervals in Fig. 2). We emphasize that other transmission mitigation decisions, such as hand washing, mask wearing, and physical distancing, were not assessed in our model. We highlight that the posterior estimates of environment and average mobility were correlated (Pearson’s r=0.30 for temperature and r=−0.32 for population density). This likely results from correlated changes in mobility and temperature through time, and makes the estimated mobility reductions in Fig. 2 conservative (i.e., we potentially report larger mobility reductions than would be necessary to mitigate environmental effects). In *SI Appendix*, we present a null model fit to data where temperature is constrained to be constant across time in all states. This null model neither fits the death data as accurately nor detects such strong impacts of temperature, suggesting that our results are not driven solely by static temperature differences across states (which may be a potential conclusion from our independent $R_{0}$ validation). Furthermore, we also present forecasting in *SI Appendix*, where we show that our model provides a good prediction of observed deaths for the 14 d following the data to which the model was fit.

## Discussion

Here, by combining epidemiological models and outputs with spatial climate data, we show that environment (specifically, cold, but also the correlated low-humidity conditions) can enhance SARS-CoV-2 transmission across the United States. Critically, however, these environmental impacts are weaker than that of population density, which is, itself, a weaker driver than policy intervention (i.e., lockdown). These results are broadly in agreement with earlier work investigating drivers of transmission in local epidemics across the United States (16). Afshordi et al. (16) found that climate (humidity, but also the highly correlated temperature) could have an impact on transmission, but that this effect was secondary to the effect of population density, which itself had a lesser effect than mobility (as a measure of nonpharmaceutical intervention strength). Our work extends this, showing that similar effects are also observed across broader spatial scales. Furthermore, our incorporation of an interaction gives the explicit finding of a minimization of the impact of environment as a driver of transmission in the presence of lockdown. Below, we suggest that the accuracy of forecasts of SARS-CoV-2 transmission, in particular across seasons, could be improved by incorporating temperature, as well as population density, in a robust, reproducible manner as we have done here.

### The Role That Environment Plays in Transmission.

**Fig. 3.**
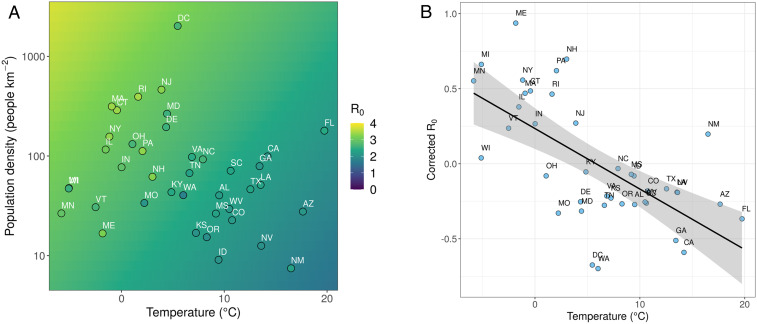
The relative importance of temperature and population density as drivers of prelockdown $R_{0}$. (*A*) Heatmap of the regression model $R_{0}$ predictions, with US state-level $R_{0}$ point estimates overlaid. High population densities and low temperatures drive increases in SARS-CoV-2 $R_{0}$. This is a 2D representation of the regression plane in Fig. 1*A*, using the same color scale. (*B*) Residuals from a linear regression of $R_{0}$ against $\log_{10}$-transformed population density (“Corrected $R_{0}$”), plotted against temperature. This illustrates that, when considering population density alone, $R_{0}$ is overestimated in cold states and underestimated in warm states. After accounting for population density, there is a significant effect of temperature upon $R_{0}$ (Table 1). In both plots, points are highlighted with standard two-letter state codes; MN and FL refer to Minnesota and Florida, respectively, and are referred to in *Discussion*.

Across these state-level US data, we found a significant negative effect of temperature on SARS-CoV-2’s $R_{0}$ and a significant positive effect of population density. An important caveat to this, however, is the collinearity between temperature, absolute humidity, and, to a lesser degree, UV levels. The strong correlations between these environmental drivers mean that we are unable to discern the effects of each in a single model, and, therefore, we focus on temperature as the most reliable environmental predictor. After accounting for the effect of population density on transmission (Table 1), temperature’s effect is striking (Fig. 3), and we speculate that some of the controversy surrounding the role environment plays in transmission may partially result from studies addressing temperature or humidity without simultaneously considering other factors such as population density. We also tested the effects of our predictor variables on $R_{t}$ for times where strict lockdown measures were in place. When these mobility restrictions are in place, we observe no significant effects of temperature on $R_{t}$; that is, the effects of lockdown dampen any environmental effects so as to make them inconsequential (Fig. 1*B* and *SI Appendix*, Table S3). Furthermore, under lockdown conditions, the overall transmission rates are vastly reduced. Through our epidemiological modeling approach, we are able to account for these effects (as mobility changes are explicitly incorporated), and find that higher population densities and lower temperatures drive increased $R_{t}$. Moreover, the formulation of our epidemiological model ensures that, under high mobility reductions, changes in environment have little effect on $R_{t}$, mirroring our regression findings (see *Materials and Methods* and *SI Appendix*, Fig. S6).

The precise physiological mechanisms for temperature-dependent inactivation in SARS-CoV-2 are still not known, but animal models for influenza have shown that increased viral transmission at lower temperatures can be due to effects on the host (6, 7). In animal models, this is proposed to be due to the combined effects of higher titers of viral particle shedding and greater viral stability in nasal passages of those housed in cooler conditions (7). In addition to host effects, the persistence time of the virus outside of the body is expected to be negatively affected by higher environmental temperatures, which cause viral inactivation via breakdown of their lipid layers (9, 11). Indeed, recent evidence suggests that SARS-CoV-2 survives for substantially longer on inert surfaces in colder conditions (39). However, both the direct host effects and the potential effects of environment on viral stability are likely moderated (if not mitigated) by indoor heating (40), although the same may not always be true of humidity. Most transmission is thought to have occurred indoors (41), increasing the potential for this mitigation factor. However, this indoor transmission study was specific to China and thus may or may not accurately represent transmission dynamics in the United States. Note also that indoor temperatures do tend to follow seasonal patterns, albeit with a lower degree of variation than outdoor temperatures (42–44). Contact rate is related to population density (15), and so it is unsurprising that population density was a significant factor in our analysis (Fig. 1*A*). We stress that temperature was not a driver of transmission under lockdown, and the effects of population density were lessened (Fig. 1*B*): Climate effects matter little when contact rates are severely diminished through policy interventions (45).

There are important methodological caveats to our findings. Dynamics and reporting between US states are known to be variable (46), introducing a level of uncertainty to our findings. Furthermore, lockdown measures were (and continue to be) quite heterogeneous across the United States, with different states displaying different levels of response to COVID-19 (47). Through our epidemiological modeling approach, we are able to account for these different state-level responses using Google mobility data. We can also observe other potential confounding factors in these analyses. Across the United States, the northeastern states in the vicinity of the major transport hub of New York City (e.g., New York, New Jersey, Maine, Pennsylvania, Rhode Island, and Connecticut) tend to have generally higher $R_{0}$ than predicted, while West Coast states (e.g., Washington, California, and Oregon) have lower $R_{0}$ than predicted (Fig. 3*A*). While this type of effect could be due to preemptive protective measures taken by states prior to COVID-19 outbreaks, we likely mitigated this by removing states that initiated nonpharmaceutical interventions before our first time step (see *Materials and Methods*). Indeed, we find no evidence of states with later COVID-19 outbreaks having lower transmission rates, showing that preemptive protective measures are unlikely to have influenced our results in any meaningful way (*SI Appendix*, Fig. S4). A further confounding factor may be seen if temperature affects human behavior, thus making it difficult to disentangle the effects of climate from changes to mobility. We do find a link between the average mobility and temperature coefficients in our Bayesian modeling, suggesting a degree of colinearity; however (perhaps surprisingly), we see no direct correlations between daily temperature and recreational mobility trends for parks (see *SI Appendix*). Again, this highlights the importance of human behavior as a confounding factor in analyses of environmental drivers on SARS-CoV-2 transmission. In the *Results*, we present data where population density is averaged across each state, which may not accurately capture any finer-scale transmission dynamics occurring within metropolitan areas. In *SI Appendix*, we explore the use of alternative metrics to capture transmission in urban populations (total population, percent population living in urban areas, and total population living in urban areas; *SI Appendix*, Table S7) but find nothing that correlates more strongly with $R_{0}$ than population density. Equally, our semimechanistic models do not explicitly measure population immigrations as a driver of transmission rates, although we could find no effect of inbound flight numbers on transmission in comparative analysis (see *SI Appendix*, Fig. S3). Nevertheless, work at smaller geographical scales (local epidemics within US counties and metropolitan areas) has produced qualitatively similar results, of climate- and population density-driven transmission, secondary to the effects of nonpharmaceutical interventions (16). We suggest that further work should be conducted at these finer spatial scales to more fully address these potential drivers of transmission intensity.

### Policy Relevance of Our Findings.

Our results comparing SARS-CoV-2 transmission rate before and during lockdown support the idea that the major driver of transmission is public health policy (34, 48, 49) (Fig. 1). Once stay-at-home measures were implemented across the United States, we can find no meaningful signal of temperature on transmission. This provides two important, and timely, insights for policy makers: Summer weather is no substitute for mitigation, and policy can prevent transmission in the winter. At the coarse scale of US states, population density is a greater driver of transmission intensity than temperature in our epidemiological modeling ($\log_{10}$ [population density] is ∼1.4× larger scaled coefficient than temperature). It should be considered whether thresholds for adaptive and/or intermittent lockdown might be more precautionary (i.e., lower) in colder, more densely populated regions. However, we strongly suggest that this should neither be in order to allow other regions to actively relax restrictions nor conducted without further examination of finer-scale disease dynamics. When making decisions about the relative importance of climate and population density, it is important to account for the magnitude of variation in the two variables. Temperature varies widely across the United States, and that differences in transmission rates between states (contrast, for example, Minnesota and Florida in Fig. 3) may vary due to climate does not imply that more-modest climate differences within a state drive differences through time or across space. Regardless, our analysis is too spatially coarse to address such variation. Even quite large variations in climate are more straightforward to mitigate than population density differences (Fig. 2), and so we suggest that regions with higher population density should continue to be monitored carefully. Finally, we emphasize that population density and temperature are well known to be strongly correlated across US states (see also Fig. 3); this does not affect our model fitting of coefficient estimates, but it does affect their interpretation. A more densely populated state is also likely to be warmer, and so we suggest that both factors (and others, such as mobility) should be taken into account when trying to a priori estimate a region’s transmission rate.

These results have strong implications for modelers considering the potential impacts of seasonality on the virus. Such work has already considered the role that seasonality might play, by assuming responses of structurally similar and/or related diseases are adequate proxies for SARS-CoV-2 (18). These assumptions are broadly correct, but here we parameterize and quantify the magnitude of this effect for SARS-CoV-2. Our findings suggest that previously unexplained variation among regions’ transmission, such as in our independently estimated $R_{0}$ data, can be accounted for by environmental factors. Further, our results support a role for daily temperature changes in transmission, but, we emphasize, do not conflict with other studies suggesting that seasonal forecasting plays a secondary role to mitigation and/or number of susceptible individuals. Such studies (18) assumed SARS-CoV-2 responds to climate to extents broadly similar to those we find here. We used the United States as a case study rather than performing a global analysis, as there are a multitude of confounding factors that may influence viral transmission rates between countries, such as wide variation in contact matrix structures (50). We are not suggesting that warmer countries cannot have high COVID-19 transmission rates; many already do. What our results do suggest, however, is that future forecasting work should consider the use of the environment to enhance predictions of disease spread. In countries such as the United States with continental climates that swing between extremes of heat and cold, we suggest policy makers should assume that transmission will increase in winter (and, potentially, autumn/fall). The timing of the seasons are broadly predictable, so this is an area in which policy could be proactive, not reactive.

### Conclusion.

There is no single cause of, or solution to, the current COVID-19 pandemic, and all drivers must be placed in perspective. Here we suggest that both environment (including population density) and daily weather may play a role in the transmission of SARS-CoV-2. However, the major driver of transmission, and our best method of controlling it, is public policy, as this and many other studies have shown (48, 49). Indeed, we have shown that, when stringent public policy measures are in place, the transmission effects of environmental drivers are negligible. Therefore, while SARS-CoV-2 may show seasonal and spatial variation in its transmission rates, these effects can be mitigated through public health interventions.

## Materials and Methods

We explored the association between environmental covariates and SARS-CoV-2 transmission intensity using two approaches. First, we took existing state-level estimates of $R_{0}$ and during-lockdown $R_{t}$ for the United States (37), and regressed them against environmental data in order to test for potential prelockdown and during-lockdown patterns. In the second approach, we modified and fitted the existing semimechanistic epidemiological model used to generate the $R_{0}$ and $R_{t}$ estimates above, and fitted it to the observed death time series while explicitly incorporating the effects of the most important aspects of environment (temperature and population density) on the virus. This second model makes use of daily weather observations and provides a rigorous framework to quantify the drivers of SARS-CoV-2 transmission across the United States. The first approach mitigates potential biases arising from the autocorrelation of the initiation of lockdown and the cessation of winter in the United States in the second approach, since our independent regression focuses on initial transmission (i.e., $R_{0}$). Below, all software packages given in *italics* are *R* packages (version 3.6.3) (51) unless otherwise specified. Code to reproduce our analyses, download source data, and update models with new data as it becomes available is given at our team’s GitHub repository (https://www.github.com/pearselab/tyrell).

### Environmental Data Collection.

We collated global population density data from the Gridded Population of the World collection (52), and hourly temperature (T), relative humidity (RH), and surface UV radiation (in joules per square meter) estimates for 2020 from the Copernicus Climate Change Service (53). All of the above data were at the same spatial resolution of 0.25 × 0.25°. The amount of water vapor air can hold increases with temperature, and since, in other viruses, the absolute humidity (AH) of air can drive transmission more than relative saturation (40, 54), we calculated absolute humidity from our data using the the Clausius–Clapeyron relation and the ideal gas law (22, 54),

$$AH=1,000\cdot \frac{e_{0}\cdot e^{\frac{L}{R_{v}}\left(\frac{1}{T_{0}}-\frac{1}{T}\right)}\cdot RH}{R_{v}\cdot T},$$ [1]

where AH (grams per cubic meter) is the absolute humidity, T (kelvin) is the temperature in a given cell, RH is the relative humidity in a given cell (expressed as a percentage), $e_{0}$ is the saturation vapor pressure (6.11 mb) at reference temperature $T_{0}$ (which we set as 273.15 K), L is the latent heat of evaporation for water ($2,257\;\text{kJ}\cdot {\text{kg}}^{-1}$), and $R_{v}$ is the gas constant for water vapor ($461.53\;\text{J}\cdot {\text{kg}}^{-1}\cdot {\text{K}}^{-1}$).

We used the Climate Data Operators program (55) to compute daily means for each of our climate variables. Finally, we averaged the value of each covariate (median) across the state-level administrative units given by shapefiles from the global administrative areas database (56) (the 50 US states, plus Washington, DC).

### Independent Validation of the Impact of Environment on $R_{0}$.

To validate the impact of the environment on $R_{0}$, we used an existing dataset of SARS-CoV-2 transmission rate estimates for each of the states of the United States (37). We used the basic reproduction estimates ($R_{0}$, before the implementation of any nonpharmaceutical interventions) as a fundamental measure of virus transmissibility in each state.

In these data, $R_{0}$ is estimated as $R_{t=0}$, where t=0 occurs 30 d prior to the first 10 cumulative deaths recorded for each state (34, 37). The date upon which $R_{0}$ is estimated therefore differs between states. To account for these temporal differences, we took the means of our daily climate variables across the 14 d prior to t=0 for each state as an approximation for the conditions under which each population first experienced COVID-19. To test the impact of the environment on $R_{0}$, we performed multiple linear regression on $R_{0}$ with temperature, absolute humidity, UV radiation, and population density as predictors. To compare environmental effects on the reproduction number under mobility restriction measures (i.e., lockdown), we took the average (mean) $R_{t}$ across the 14 d following a state-wide stay-at-home mandate and regressed these against the environmental predictors averaged across the same time period. We used 14 d again here for consistency with our environmental comparison to $R_{0}$. Although mobility restrictions may differ in magnitude between states, these effects are incorporated into the estimates for the $R_{t}$ parameter. In seven states (Arkansas, Iowa, North Dakota, Nebraska, Oklahoma, South Dakota, and Wyoming), no state-wide stay-at-home mandate was declared. In a further four states (Alaska, Hawaii, Montana, and Utah), t=0 occurred after nonpharmaceutical interventions had already been instated. These 11 states were therefore excluded from the independent validation analyses.

### Integrative Modeling of the Impact of Environment on SARS-CoV-2 Transmission.

To further assess the potential impact of environment on SARS-CoV-2 transmission, we modified the semimechanistic Bayesian model (37) that generated the $R_{t}$ estimates used above to incorporate both population density and daily temperature (the best-fitting climate variable; see *Results*). Full details of the model are given in Unwin et al. (37) and are replicated in *SI Appendix*, *Bayesian Model*). Below, we briefly outline the model’s structure and then emphasize the changes we have made. Unwin et al. (37) fit their model to daily deaths across the United States in each state, using the working assumption that death data are more complete than case incidence data. Each biogeographic region has its own infection–fatality ratio (IFR) estimate based on previously estimated IFRs from China (57) that have been scaled to reflect the population demographics of each state, and contact patterns of the United Kingdom (the behavior assumed closest to the United States from the available data). These estimates form the mean value of the IFR in the model, with normally distributed estimated variation. The time from infection until death is also modeled using data from previous empirical studies as a mixed distribution [Γ(5.1,0.86)+Γ(17.8,0.45)] in order to account for uncertainty in the time from infection to symptom onset and the time from symptom onset to death. We changed the estimation of transmission across states from (37) as follows:

$$\begin{array}{c} R_{t,m}=\left(\mu +c_{t,m}C+p_{m}P\right)\cdot 2InvLogit\left(-\sum \left(X_{t,m,k}\alpha_{k}\right)-X_{t,m,1}\alpha_{r\left(m\right)}^{region}-X_{t,m,2}\alpha_{m}^{state}-\epsilon \right), \end{array}$$ [2]

where μ captures overall transmission common to all states, C is the coefficient for temperature ($c_{t,m}$; in degrees Celsius) at time (t) in state m, and P is the coefficient for population density ($p_{m}$) of state m ($\log_{10}$-transformed people per ${km}^{2}$). We standardized $c_{t,m}$ and $p_{m}$ to have a mean of zero and standard deviation of one, in order to make their absolute magnitudes measures of the relative importance of each term and thus facilitate their comparison. We placed strong, conservative priors on these new model terms, specifically,

$$c_{t,m},p_{m}∼Normal\left(0,0.5\right)$$ [3]

μ∼Normal(3.28,0.5). [4]

For μ, this is the same as the prior used in the original (nonclimate) model (37) (but see our caveat below about this term). The other terms are unchanged from their original definitions given in ref. 37, and we briefly describe them below. InvLogit is the inverse logit transformation applied to a series of hierarchically nested terms ($\alpha_{k}$, $\alpha_{r\left(m\right)}^{region}$, and $\alpha_{m}^{state}$) multiplied by Google mobility data (38) ($X_{t,m,k}$) with a weekly AR(2) autocorrelated error term for each state (ϵ; see ref. 37 and *SI Appendix* for more details). $X_{t,m,k}$ are three US-wide measures of the impact of changing mobility across states on “average” across retail and recreation, grocery and pharmacy, and workplace trips ($X_{t,m,1}$), in “residential” areas ($X_{t,m,2}$), and using public “transit” ($X_{t,m,3}$). These mobility data show changes in individuals’ behavior following government interventions and thus represent a daily proxy for lockdown intensity (and concomitant impacts on contact rates). We focus on the vector $\alpha_{k}$, whose three entries assess the impact of mobility comparably across the country (and thus are each analogous to c and p). The terms $\alpha_{r\left(m\right)}^{region}$ and $\alpha_{m}^{state}$ address differences in average mobility across eight broad geographic regions (the Great Lakes, Great Plains, Rocky Mountains, Northeast Corridor, Pacific Northwest, South Atlantic, Southern Appalachia, and the South; indexed by r(m)) and for transit across individual states (m), respectively. While we attempted to address comparable hierarchically nested temperature responses in this model, we felt the correlation between changes in $X_{t,m,k}$ and $c_{t,m}$ was inducing fitting problems and so opted for a model simpler (and so more conservative) in its novel components. In this model formulation, temperature and population density essentially contribute to a latent transmission rate, which is then mediated by the mobility terms to produce the realized $R_{t}$. Although an interaction between mobility and environment (as found in our regression modeling; see *Results*) is not explicitly modeled, this formulation produces results analogous that finding; that is, when mobility reductions are high (“lockdown”), environment has little effect on the realized $R_{t}$ (see *SI Appendix*, Fig. S6).

We emphasize that the model presented here differs from the original model by fitting a common μ across all states, instead of allowing each state to have a different baseline μ that was hierarchically drawn from a common parameter (itself termed μ in ref. 37). This difference ensures identifiability of our model parameters, since the (latent, and hierarchically pooled) state-wise means are strongly driven by both population density and environment that are now included in the model (see *Results*). Our model, which was directly adapted from the code in ref. 37, was fit using *rstan* (58) with five independent chains (each with 3,000 total iterations and a warm-up of 1,000). Full model coefficients and outputs are given in *SI Appendix*, Table S6; posterior predictive checks were performed to ensure that the predicted $R_{t}$ values for each state through time were realistic and sensible and that all chains had mixed and converged.

We performed additional analyses to cross-validate our model findings. First, we used fitted coefficients with known temperature and mobility data to forecast deaths into the 14 d immediately following the date range that the model was fitted to. We also compared the predicted deaths to the observed deaths over the same time period to validate the accuracy of our model. These predictions were conducted using a separate cross-validation run of our model (five chains of 1,500 iterations with a warm-up of 1,000). Secondly, we validated the inclusion of daily temperature change in our model by comparing the fit to that of a null model where temperature remains constant through time. In this null model, we replaced all daily temperature within a state with that state’s median temperature across the study period, and fit the model as described above. Thus this null model tests whether it is the average temperature of a state, rather than daily temperature, that drives changes in transmission through time. We tested this by asking whether the effect of temperature is as strong as in our original model (i.e., the magnitude of the temperature coefficient, C); if not, this validates our inclusion of daily temperature change in our model.

## Supplementary Material

Supplementary File

## Data Availability

No new data are released as part of this paper, all external datasets used are publicly available, described in *Materials and Methods* and referenced. Outputs from our Bayesian model runs are available on Figshare (https://doi.org/10.6084/m9.figshare.14696841.v1) and code to reproduce our analyses are available on Zenodo (https://doi.org/10.5281/zenodo.4884696). Code to download and combine external datasets and reproduce our full analysis pipeline is also available in our team’s GitHub repository (https://www.github.com/pearselab/tyrell).
