# Supplementary material for: Temperature and population density influence SARS-CoV-2 transmission in the absence of nonpharmaceutical interventions
Source: Proc Natl Acad Sci U S A. 2021 Jun 8;118(25):e2019284118. doi: 10.1073/pnas.2019284118 (PMC8237566; doi:10.1073/pnas.2019284118)
Supplement: Supplementary File [file pnas.2019284118.sapp.pdf]

PNAS
www.pnas.org

Supplementary Information for

Temperature and population density influence SARS-CoV-2 transmission in the absence of non-pharmaceutical interventions

Thomas P. Smith, Seth Flaxman, Amanda S. Gallinat, Sylvia P. Kinosian, Michael Stemkovski, H. Juliette T. Unwin, Oliver J. Watson, Charles Whittaker, Lorenzo Cattarino, Ilaria Dorigatti, Michael Tristem, William D. Pearse

Thomas P. Smith & William D. Pearse.
E-mail: thomas.smith1@imperial.ac.uk or will.pearse@imperial.ac.uk

This PDF file includes:

Supplementary text
Figs. S1 to S8
Tables S1 to S8
SI References

Supporting Information Text

Data and Code Availability

The entirety of the code to reproduce our analyses, download source data and update models with new data are available from our *GitHub* repository (https://www.github.com/pearselab/tyrell). We also provide releases of the R workspaces from the Bayesian model runs used in the manuscript: the main model, the cross-validation forecasting model and cross-validation null model.

Collinearity of Climate Variables

As stated in the main text, correlations between the climate variables (temperature, absolute humidity, UV radiation levels) mean that we could not disentangle each variable's contributions to R_0 in multiple regression analysis. This can be seen in the high variance inflation factors for all climate variables when these predictors are regressed together (table S1).

To further illustrate this, we perform a principal components analysis (PCA) on the three climate variables in our dataset, plus population density. Eigenvalues of the first two components of the combined datasets together accounted for 83% of the total variance. Temperature and absolute humidity fall mainly along the axis of PC1, which accounts for 55% of the variation. This close relationship is expected given that absolute humidity is a function of temperature (see main text eq. 1). UV radiation is correlated with temperature and humidity, but less so, and contributes more to PC2 which also comprises population density and explains 28% of the variance (fig. S1).

For further analysis we therefore chose the best fitting climate variable, assessed by Pearson's r (table S2). Based on this, we used temperature as our climate variable in further analyses.

Effects of environment before and during lockdown

When regressed against temperature and $\log_{10}$-transformed population density only, we find that R_0 significantly increases with population density and decreases with temperature (both $p < 0.001$, table S3, see main text Fig. 1A and fig S2). However, when regressing lockdown R_t (mean R_t across the two weeks following a state-wide stay-at-home mandate) against temperature and $\log_{10}$-transformed population density, we find no significant effect of temperature, and a much lower coefficient for population density than in the R_0 regression model (table S4).

Lockdown as a model term. We combine the pre-lockdown R_0 and during-lockdown R_t data for the USA and perform multiple linear regression with lockdown as an additional parameter, to further investigate the impact of mobility restrictions (*i.e.* lockdown) compared to the environment. We restrict the environmental parameters to temperature and population density due to the collinearity between temperature, UV radiation and humidity (see section 1). We first incorporate lockdown as a binary (*i.e.* in lockdown, or not in lockdown) additive effect and test the effects on R:

$$R \sim Temperature + \log_{10}(Population\ density) + Lockdown$$

As expected, we find that lockdown has a significant effect on R, with an order of magnitude greater strength than either environmental parameter (table S5).

Subsequently we ask whether lockdown mediates the environmental effects by incorporating this as an interaction term, *i.e.*:

$$R \sim Temperature + \log_{10}(Population\ density) + Lockdown + \\ Temperature : Lockdown + \log_{10}(Population\ density) : Lockdown$$

Here we find that there is a significant interaction between lockdown and temperature, but not population density (table S6), *i.e.* the effects of temperature on R are dependant upon lockdown status. This interaction model is preferred over the purely additive model (ANOVA, $F_{2,74} = 9.996$, $p < 0.001$). This is the result presented in the main text.

Additional explanatory variables

Urban population. Whilst we have shown an effect of state-level population density on R, this may not be fully representative of the transmission dynamics if most transmission occurs within metropolitan areas. We therefore explored the use of census data specifying the proportion of people living in urban environments as an alternative predictor to population density. We also considered the total population of each state and the total urban population of each state (*i.e.* % urban population $\times$ total population). However, we find that population density is by far the strongest correlate of R_0 (table S7), and therefore use this as the population demographic predictor in our models.

Airport arrivals. Our modelling approach accounts for the comparative movement of people within states using the mobility data from Google, both for our independent validation R estimates, as well as within the Bayesian model that we have adapted here. However, we have no variables specifically taking into account visitors from other states or countries, which may increase a state's transmission rate. To investigate this potential effect, we asked whether R_0 increases in states with a great amount of air traffic. We collated data from the Federal Aviation Administration's performance metrics giving daily flight arrivals at 77 of the USA's busiest airports (the ASPM77). We compared the cumulative arrivals in the two weeks proceeding our R_0 estimate by state, but find no correlation with R_0. Furthermore, the states with the greatest numbers of airport arrivals have comparatively low R_0 estimates (Florida, California, Texas, Fig. S3). Note however that we cannot differentiate between inter- and intra-state flight arrivals here.

Potential confounding factors

Epidemic starting dates. For each state, R_0 was estimated 30 days prior to the first 10 cumulative deaths recorded (see methods). This means that R_0 was estimated on different dates for each state, depending upon when the epidemic took hold. However, if people in states where the epidemic took hold later had already begun to modify their behaviours based on the situation in earlier succumbing states, this may result in a reduction in R_0 in those later states. Moreover, if there were spatial component to this, *i.e.* epidemic beginning earlier in Northern (colder) states and later in Southern (warmer) states, it could result in a spurious correlation between temperature and R_0. We investigated this potential confounding factor, but find no relationship between R_0 and the date it was estimated on (Fig. S4). Furthermore, we see no relationship between these dates and the temperatures across states, *i.e.* epidemics did not generally take hold in colder states and transfer to warmer states later (but note the strong partitioning in R_0 between colder and warmer states, Fig, S4). Therefore, the lower transmission rates seen in warmer states are not due to epidemics starting later in these states and giving the population the opportunity to modify their social behaviour in preparation.

Effect of environment on recreational mobility. Mobility trends for parks were omitted from estimates of R_t both from the datasets that we used our external validation exercise, as well as our own Bayesian modelling (as significant contact events are assumed to be negligible). However, here we further investigate the impacts of environmental temperature on outdoor recreational mobility levels as a potential confounding factor in these types of analysis (*i.e.* "do people go to the park more when it's warm?"). We first detrend the data using the diff() function in R, as the effects of policy are expected to have a large impact on the realised mobility levels. This allows us to compare whether an increase in environmental temperature at the daily level causes a similar increase in recreational mobility. When we compare daily changes in mobility against daily changes in temperature across all US states, between February and June 2020, we find no significant correlation ($t_{5276} = -0.64628$, $r = -0.0089$, $p = 0.518$).

Bayesian model

Model structure. The model used to generate R_0 and R_t estimates used in our independent validation of environmental effects is presented in Unwin *et. al.* (1). This model is based on the framework provided by Flaxman *et. al.* (2), in which R_t informs a latent function for infections, which are then calibrated against observed deaths, but has been adapted to paramaterise R_t as a function of google mobility data.

Specifically, the number of infected individuals, i, is modelled using a discrete renewal process. The number of infections $i_{t,m}$ on a given day t, and state m, is given by the following function:

$$\begin{aligned} i_{t,m} &= S_{t,m} R_{t,m} \sum_{\tau=0}^{t-1} i_{\tau,m} g_{t-\tau}, \\ S_{t,m} &= 1 - \frac{\sum_{j=0}^{t-1} i_{j,m}}{N_m}, \end{aligned} \quad [1]$$

where the population of state m is denoted by N_m and the adjustment factor $S_{t,m}$ accounts for the number of susceptible individuals in the population. The generation distribution g is discretised by $g_s = \int_{s-0.5}^{s+0.5} g(\tau)d\tau$ for $s = 2, 3, ...$ and $g_1 = \int_0^{1.5} g(\tau)d\tau$, where g has a density $g(\tau)$ of:

$$g \sim \Gamma(6.5, 0.62) \quad [2]$$

Daily deaths, $D_{t,m}$, are defined for days $t\epsilon\{1, ..., n\}$ and states $m\{1, ..., M\}$ and modelled using a function $d_t m = \mathbb{E}[D_{t,m}]$ that represents the expected number of deaths attributed to COVID-19. $D_{t,m}$ is assumed to follow a negative binomial distribution with mean $d_{t.m}$ and variance $d_{t,m} + \frac{d^2_{t,m}}{\psi_1}$, where ψ follows a positive half normal distribution.

Observed deaths are mechanistically linked to transmission, using previously estimated COVID-19 infection fatality ratios (IFRs), where each state has a specific mean IFR (ifr_m). The distribution of times from infection to death, π was assumed (based on previous epidemiological studies) to be a convolution of an infection-to-onset distribution ($\pi\prime$) and an onset-to-death distribution:

$$\pi \sim \Gamma(5.1, 0.86) + \Gamma(17.8, 0.45). \quad [3]$$

The expected number of a deaths $d_{t,m}$ on a given day t, for a given state m, is given by:

$$d_{t,m} = \text{ifr}^*_m \sum_{\tau=0}^{t-1} i_{\tau,m} \pi_{t-\tau}, \quad [4]$$

where $i_{\tau,m}$ is the number of new infections on day τ in state m and π is discretised via $\pi_s = \int_{s-0.5}^{s+0.5} \pi(\tau)d\tau$ for $s = 2, 3, ...$ and $\pi_1 = \int_0^{1.5} \pi(\tau)d\tau$ where $\pi(\tau)$ is the density of π.

$R_{t,m}$ is parameterised as a function of the relative change in human mobility from a baseline:

$$R_{t,m} = R_{0,m} \cdot f\left(-\left(\sum_{k=1}^{2} X_{t,m,k}\alpha_k\right) - \sum_{l=1}^{2} Y_{t,m,l}\alpha^{region}_{r(m),l} - Z_{t,m}\alpha^{state}_m - \epsilon_{m,w_m(t)}\right), \quad [5]$$

where $f(x) = 2exp(x)/(1+exp(x))$ is twice the inverse logit function. $X_{t,m,k}$ are covariates that have the same effect for all states, $Y_{t,m,l}$ is a covariate that has region-specific effects, $Z_{t,m}$ is a covariate with state-specific effects and $\epsilon_{m,w_m(t)}$ is a weekly AR(2) processed which captures the variation between states not explained by the mobility covariates. The mobility covariates used were: $X_{t,m,1} = M^{average}_{t,m}$, $X_{t,m,2} = M^{residential}_{t,m}$, $Y_{t,m,1} = 1$ (an intercept), $Y_{t,m,2} = M^{average}_{t,m}$ and $Z_{t,m} = M^{average}_{t,m}$, where $M^{average}_{t,m}$ is the average change in mobility for retail and recreation, groceries and pharmacies, and workplaces, and $M^{residential}_{t,m}$ is the change in mobility for places of residence.

The prior distribution for $R_{0,m}$ was chosen to be:

$$R_{0,m} \sim N(3.28, k) \text{ with } k \sim N^+(0, 0.5), \quad [6]$$

where κ is the same among all states. Seeding of new infections was assumed to begin 30 days before first 10 cumulative deaths recorded by a state. From this date, the model was seeded with 6 sequential days of an equal number of infections $i1, m = ... = i_{6,m} \sim$ Exponential($\frac{1}{\tau}$), where $\tau \sim$ Exponential(0.03). The seed infections are inferred in the Bayesian posterior distribution.

The prior distribution for the shared coefficients are given as:

$$\alpha_k \sim N(0, 0.5), k = 1, ..., 3, \quad [7]$$

and the prior distributions for pooled coefficients as:

$$\alpha^{region}_{r,l} \sim N(0, \gamma_r), r = 1..., R, l = 1, 2 \text{ with } \gamma_r \sim N^+(0, 0.5), \quad [8]$$

$$\alpha^{state}_m \sim N(0, \gamma_s), r = m..., M \text{ with } \gamma_s \sim N^+(0, 0.5). \quad [9]$$

Parameters were jointly estimated for all states in a single hierarchical model, with fitting performed in the Stan programming language.

To integrate temperature and population density into this model, we made a number of alterations to the model structure, see main text methods.

Model coefficients. Figure S5 shows the posterior interval estimates from MCMC draws for the main parameters in our Bayesian model. We also provide the full model coefficients and results of posterior predictive checks to ensure that all chains had mixed and converged in table S8.

Contribution of Bayesian model terms to R_t. We provide a visualisation showing the influence of our various model terms on R_t for each state through time in fig S6. When reductions in mobility are strong, R_t is largely driven by the mobility terms in the model, however when mobility reductions are weak, temperature and population density are much greater drivers of R_t. See for example New York: when mobility was close to baseline in February and March, fluctuating temperatures cause large spikes in R_t, which is also generally driven upwards by the relatively high population density of the state. However, after stay-at-home measures were implemented, R_t drops and closely tracks changes in mobility, with temperature fluctuations having much lesser an effect. This shows that although we don't explicitly model an interaction between environment and mobility, our model represents this effect.

Cross-validation. To validate the point-estimates of our model, we use our fitted coefficients with known temperature and mobility data to forecast deaths into the 14 days immediately following the date range that the model was fitted to. Our model provides a good prediction over this 14-day period, with predicted deaths highly correlated with the real observed deaths (Pearson's $r = 0.99$, Fig. S7). We visualise these predictions for each state in Fig. S8, where the time series of death data is shown with the model predictions overlaid. Whilst the model provides a good prediction of deaths over this period, we can observe that the uncertainty in the predictions rapidly increases (wide credible intervals) over the prediction range. This is in line with previous validation of the base model used here, which found good forecasting over short-timescales, but that performance degrades rapidly at longer time-scales (2).

To further validate the inclusion of daily temperature variation our model, we compared the model fit to that of a null model in which we replaced all daily temperature data within a state with that state's median temperature, and fit the model as described in the methods. This allows us to ask whether the daily changes in temperature through time are an important factor, or whether only the absolute differences in temperature between states are important (*i.e.* the temperature-R_0 correlation in our independent regression analyses). Within this model run, the posterior distribution for C suggested limited-to-no residual effect of temperature (median -0.038; 95% CIs -0.26 – $+0.23$). This supports our conclusion that changes in temperature and transmission are correlated through time: it is not the average temperature of a state through the four-month period of this study that is associated with daily transmission, but rather the temperature on each individual day. Were our findings driven solely by average temperature differences across US states, and not daily temperature, we would have found similar coefficient estimates between our observed data and this null analysis.

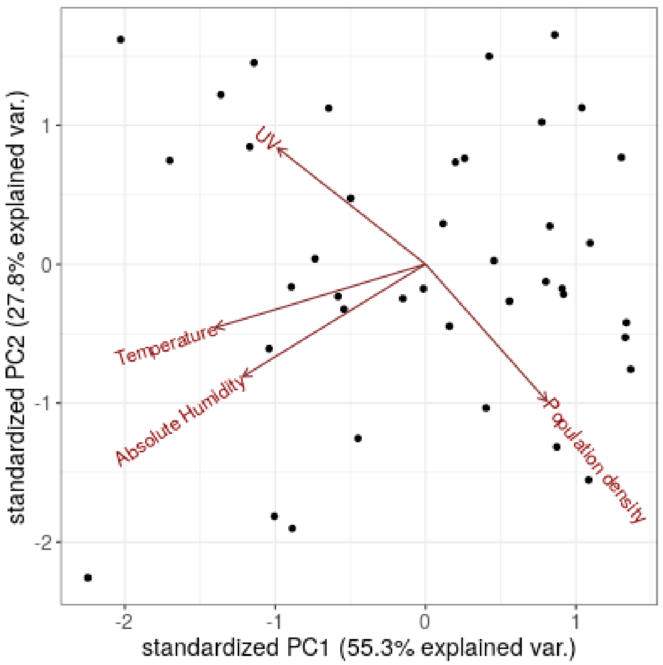

Fig. S1. **Results of PCA (PC1 vs PC2) carried out on climate variables and population density for each state.** Arrows correspond to each of the analysis variables. The proportion of variance explained by each axis is shown on the axis labels. The climate variables condense mainly into PC1, whilst population density explains more of the variance in PC2.

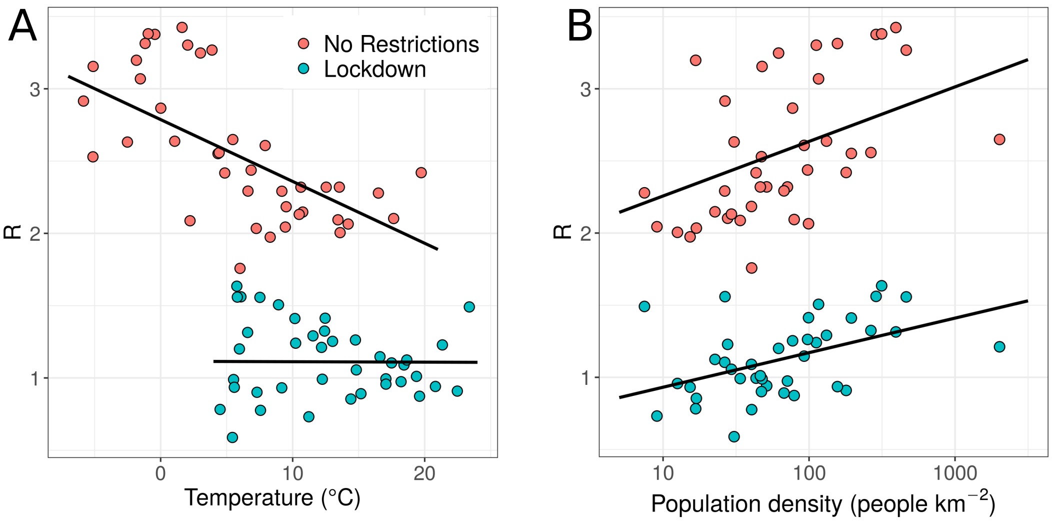

Fig. S2. **Impacts of the environment and lockdown on R.** This figure gives 2D representations of our regression model findings for the effects of temperature (A) and population density (B) on R, both pre-lockdown (red) and during-lockdown (blue). Lines give 2D representations of the predicted R from the regression model (table 1, main text), using the median population density across all states for the temperature figure, and the median temperature across states for the population density figure, for pre- and during-lockdown respectively.

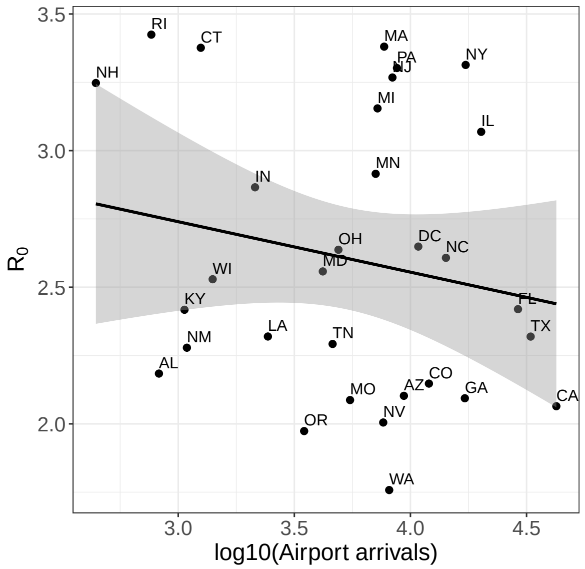

Fig. S3. $\mathbf{R_0}$ **estimates compared to airport arrivals.** Each point is the R_0 estimate for each state, versus the cumulative inbound flights in the two weeks proceeding the R_0 estimate. There is no association between flight arrivals and R_0 (linear regression, black line with grey confidence envelope, $p = 0.31$). Arrivals are $\log_{10}$ transformed as these data are heavily right-skewed. This is based on data from the 77 busiest airports in the USA; some states have no airports within this dataset and so are omitted here.

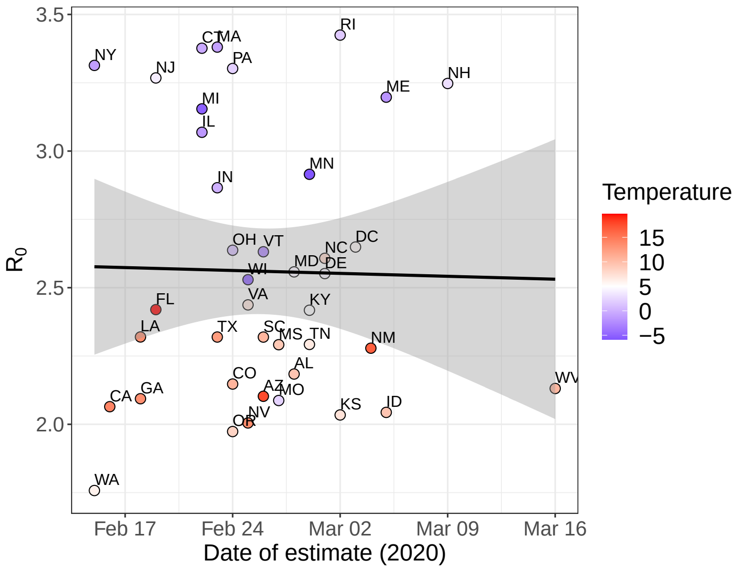

Fig. S4. R_0 **estimates compared to the dates they were estimated upon.** Each point is the R_0 estimate for each state, and the date upon which it was estimated, coloured by the average temperature over the two weeks prior to this date (see methods). There is no association between R_0 and date (linear regression, black line with grey confidence envelope, $p = 0.91$).

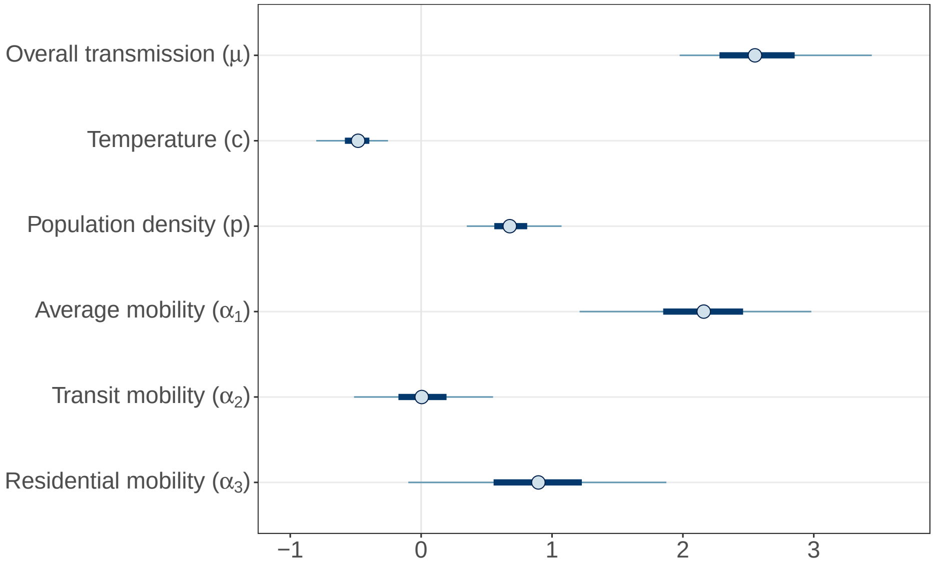

Fig. S5. Bayesian parameter distributions. Here we show the uncertainty intervals from posterior draws for the main parameters in our epidemiological model. These are the overall transmission rate common to all states, the three measures of mobility, and the temperature and population density parameters that we added to the original model (each with their symbols as given in equation 2 [methods] in parentheses). Point represents median, thick line 50% interval, thin line 95% interval.

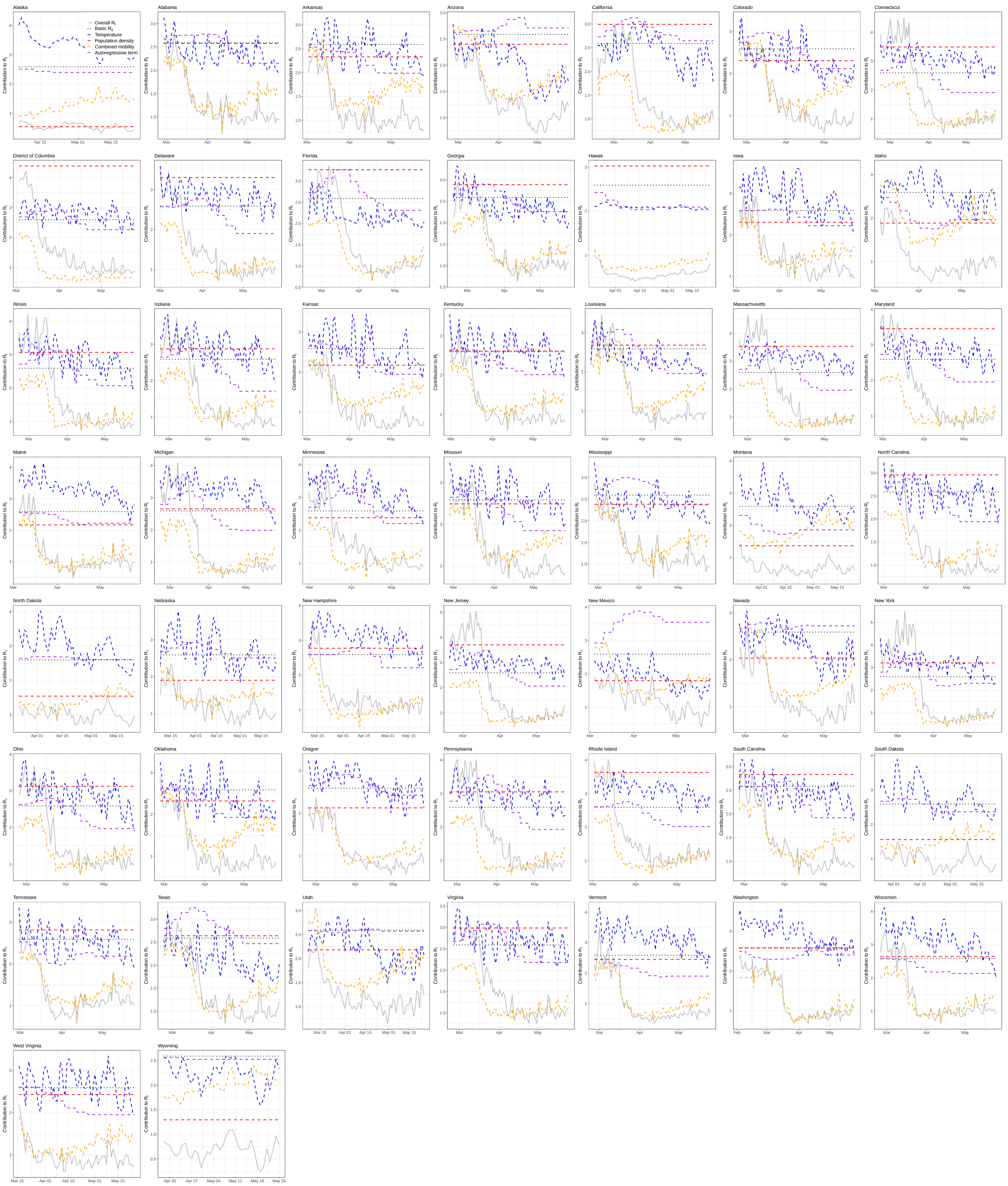

Fig. S6. Relative contribution of model terms driving changes in R_t through time. The black dotted line shows the "basic R_0" term, which is constant through time for all states. The dashed lines represent temperature (blue), population density (red), combined mobility reductions (orange) and the autoregressive term (purple) and show what R_t would be estimated at if the model only considered each of these terms alone. The grey soild line shows the realised R_t estimated by the model. Essentially this shows the relative contribution of the model terms in pulling R_t away from the baseline R_0 (black dotted) to the realised R_t (grey soild).

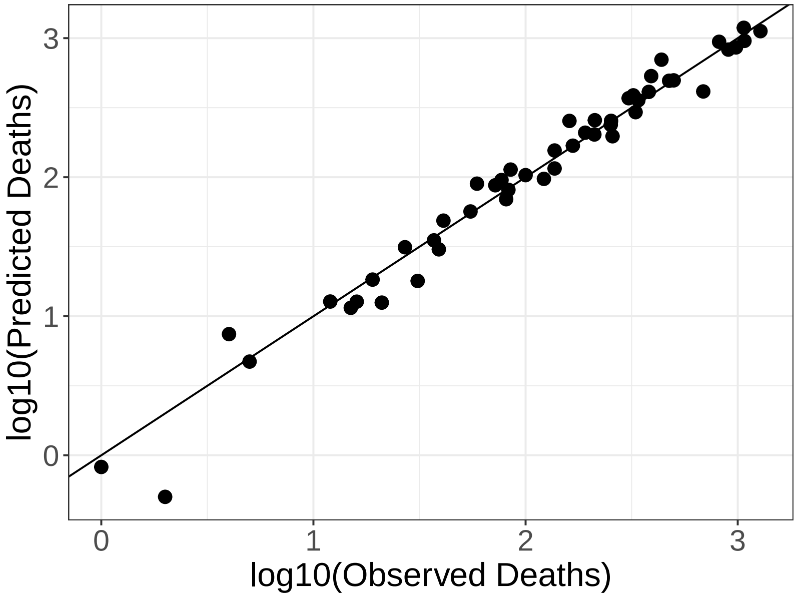

Fig. S7. Observed vs predicted deaths. Here we show the cumulative predicted deaths for a each state over the 14-day prediction period, plotted against the real observed deaths over the same period ($\log_{10}$ transformed). Black line is 1:1. Observed deaths are highly correlated with those predicted by our model (Pearson's $r = 0.99$). Two states with zero observed deaths over this period (AK and HI) are omitted from this plot, although their lack of deaths were also well predicted by the model (median cumulative death predictions for AK = 0.28, HI = 0.34).

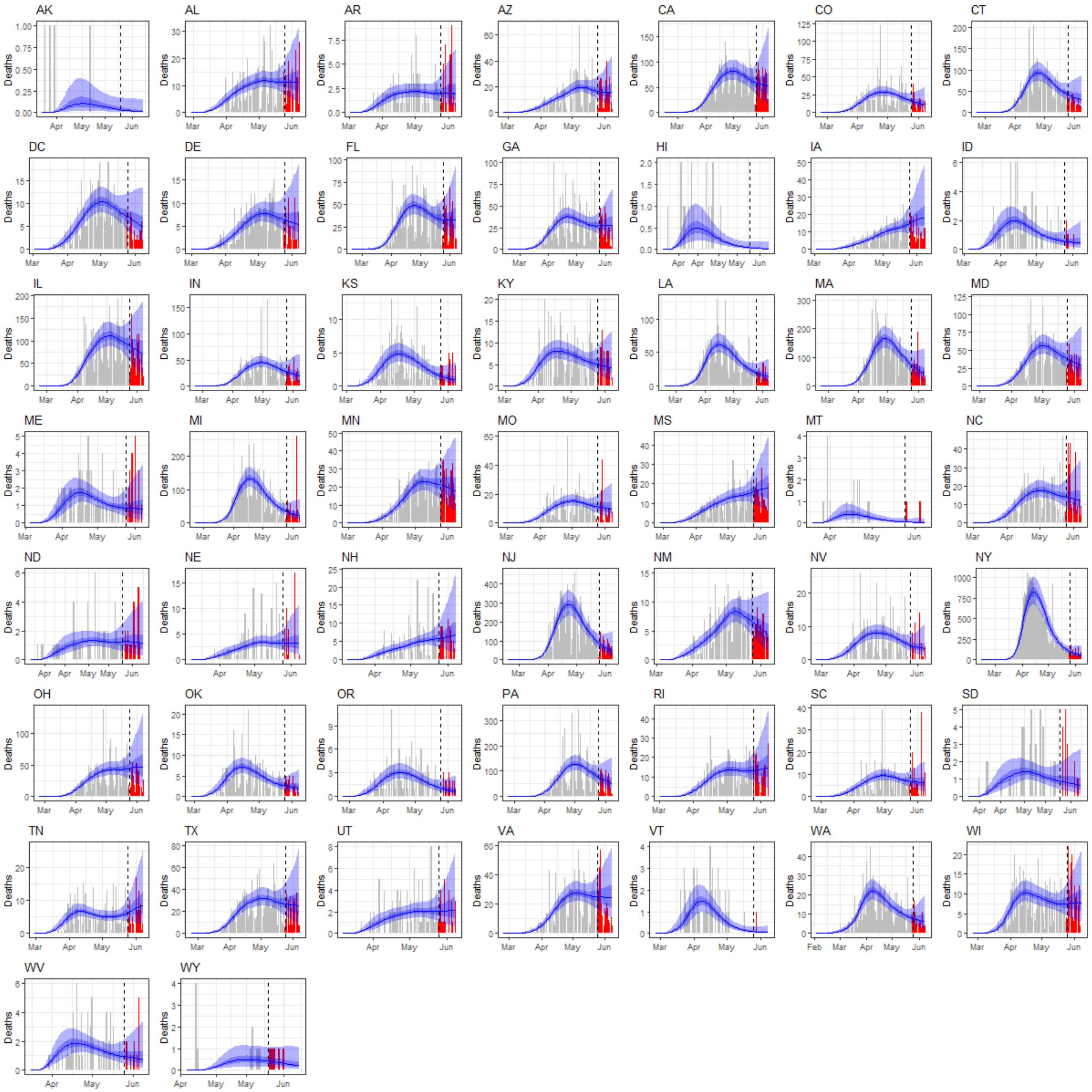

Fig. S8. Predicted deaths by state. Here we show the observed and predicted deaths for each state across the entire time-series of our model. Grey bars show the daily recorded deaths for the date range that our model is fitted to. Red bars show the observed daily deaths in the range which our model is predicted for without death data (with the dashed line designating the split between these data ranges). Blue line is the median death prediction, with dark blue (50%) and light blue (95%) credible interval bands for the predictions.

Table S1. Multiple regression with all climate variables results in high inflation of variance.

	Scaled Estimate	Std. Error	t value	p-value	VIF
(Intercept)	2.56	0.050	51.6	< 0.001*	-
Temperature	-0.39	0.129	-3.04	0.0045*	6.61
Abs. Humidity	0.09	0.116	0.75	0.46	5.30
UV radiation	0.10	0.075	1.30	0.20	2.24
$\log_{10}$(Pop density)	0.22	0.057	3.91	< 0.001*	1.29

$r^2 = 60\%$, $F_{4,38} = 13.96$, $p < 0.001$. Scaled estimates are coefficients when predictors are scaled to have mean = 0 and SD = 1. The variance of environmental parameters are inflated due to their strong collinearity (VIF = variance inflation factor). $* = p < 0.05$

Table S2. Assessment of the best fitting climate variable.

Variable	Pearson's r
Temperature	-0.675
Absolute humidity	-0.561
UV	-0.357

Comparison of correlation coefficients between R_0 and each of the climate variables; temperature, absolute humidity and surface UV levels. Temperature shows the highest correlation with R_0.

Table S3. Population density and temperature are drivers of R_0 at state-level in the USA.

	Scaled Estimate	Std. Error	t value	p-value
(Intercept)	2.56	0.049	51.8	< 0.001*
Temperature	-0.28	0.052	-5.43	< 0.001*
$\log_{10}$(Pop density)	0.19	0.052	3.72	< 0.001*

$r^2 = 60\%$, $F_{2,37} = 28.2$, $p < 0.001$. Scaled estimates are coefficients when predictors are scaled to have mean = 0 and SD = 1. Scaling our explanatory variables means our coefficients are measures of the relative importance of each variable. In contrast to our epidemiological modelling, temperature is a greater driver of pre-lockdown R_0 than population density ($\log_{10}$-transformed). * = $p < 0.05$

Table S4. Lockdown reduces the effects of environment on R.

	Scaled Estimate	Std. Error	t value	p-value
Intercept	1.13	0.037	30.1	< 0.001*
Temperature	-0.002	0.039	-0.04	0.967
$\log_{10}$(Pop density)	0.12	0.039	3.10	0.004*

$r^2 = 22\%$, $F_{2,37} = 5.21$, $p = 0.01$. Scaled estimates are coefficients when predictors are scaled to have mean $= 0$ and SD $= 1$.

Table S5. Lockdown has a stronger effect on R than environment.

	Scaled Estimate	Std. Error	t value	p-value
(Intercept)	2.47	0.053	46.9	< 0.001*
Temperature	-0.18	0.041	-4.25	< 0.001*
$\log_{10}$(Pop density)	0.15	0.036	4.23	< 0.001*
Lockdown	-1.26	0.080	-15.8	< 0.001*

$r^2 = 86\%$, $F_{3,76} = 160.5$, $p < 0.001$. Scaled estimates are coefficients when predictors are scaled to have mean = 0 and SD = 1.

Table S6. Lockdown conditions mediate the effects of environment on R.

	Scaled Estimate	Std. Error	t value	p-value
(Intercept)	2.41	0.050	48.3	< 0.001*
Temperature	-0.30	0.049	-6.13	< 0.001*
$\log_{10}$(Pop density)	0.19	0.045	4.20	< 0.001*
Lockdown	-1.29	0.072	-17.8	< 0.001*
Temperature:Lockdown	0.30	0.075	3.92	< 0.001*
$\log_{10}$(Pop density):Lockdown	-0.07	0.064	-1.09	0.29

$r^2 = 89\%$, $F_{5,74} = 123.1$, $p < 0.001$. Scaled estimates are coefficients when predictors are scaled to have mean = 0 and SD = 1.

Table S7. Correlations of population demographic predictors with R_0.

Predictor	Pearson's r
$\log_{10}$(Population density)	0.54
$\log_{10}$(Total population)	-0.04
% Urban population	0.08
$\log_{10}$(Total urban population)	-0.02

Data were $\log_{10}$ transformed when strongly right-skewed.

	mean	se_mean	sd	2.5%	25%	50%	75%	97.5%	n_eff	$\hat{r}$
α_1	2.14	0.01	0.45	1.21	1.85	2.16	2.46	2.98	1394.56	1.00
α_2	0.01	0.01	0.27	-0.51	-0.17	0.00	0.19	0.55	2401.50	1.00
α_3	0.89	0.01	0.50	-0.10	0.55	0.90	1.23	1.87	3778.68	1.00
α_1^{state}	-0.00	0.00	0.31	-0.67	-0.11	-0.00	0.11	0.66	11062.97	1.00
α_2^{state}	0.00	0.00	0.29	-0.63	-0.11	0.00	0.11	0.66	13676.39	1.00
α_3^{state}	0.00	0.00	0.30	-0.65	-0.11	0.00	0.12	0.65	12106.48	1.00
α_4^{state}	0.00	0.00	0.30	-0.64	-0.10	0.00	0.11	0.65	12138.84	1.00
α_5^{state}	-0.04	0.00	0.27	-0.67	-0.14	-0.01	0.07	0.49	4508.57	1.00
α_6^{state}	0.00	0.00	0.29	-0.61	-0.11	0.00	0.11	0.64	14340.61	1.00
α_7^{state}	0.00	0.00	0.30	-0.65	-0.11	0.00	0.11	0.66	13683.76	1.00
α_8^{state}	-0.02	0.00	0.24	-0.56	-0.12	-0.00	0.09	0.50	6369.54	1.00
α_9^{state}	-0.00	0.00	0.30	-0.65	-0.11	-0.00	0.11	0.64	11822.21	1.00
α_{10}^{state}	-0.00	0.00	0.30	-0.67	-0.11	-0.00	0.10	0.64	13655.71	1.00
α_{11}^{state}	-0.00	0.00	0.31	-0.67	-0.12	-0.00	0.11	0.66	12288.08	1.00
α_{12}^{state}	-0.00	0.00	0.30	-0.66	-0.11	-0.00	0.11	0.63	10459.35	1.00
α_{13}^{state}	0.00	0.00	0.29	-0.65	-0.11	-0.00	0.11	0.63	11717.81	1.00
α_{14}^{state}	0.00	0.00	0.30	-0.65	-0.11	-0.00	0.11	0.65	11918.50	1.00
α_{15}^{state}	-0.00	0.00	0.26	-0.56	-0.11	0.00	0.10	0.57	7866.88	1.00
α_{16}^{state}	-0.00	0.00	0.31	-0.68	-0.11	-0.00	0.11	0.66	12413.47	1.00
α_{17}^{state}	-0.00	0.00	0.30	-0.66	-0.11	-0.00	0.11	0.66	12689.82	1.00
α_{18}^{state}	-0.00	0.00	0.30	-0.68	-0.11	0.00	0.11	0.66	15471.48	1.00
α_{19}^{state}	0.00	0.00	0.29	-0.64	-0.10	0.00	0.11	0.65	12943.23	1.00
α_{20}^{state}	-0.03	0.00	0.25	-0.61	-0.14	-0.01	0.07	0.48	4880.30	1.01
α_{21}^{state}	0.00	0.00	0.26	-0.56	-0.10	-0.00	0.10	0.57	6802.89	1.00
α_{22}^{state}	0.00	0.00	0.29	-0.64	-0.11	0.00	0.11	0.64	14606.26	1.00
α_{23}^{state}	-0.00	0.00	0.30	-0.66	-0.11	-0.00	0.11	0.66	12520.77	1.00
α_{24}^{state}	-0.00	0.00	0.30	-0.66	-0.11	-0.00	0.10	0.66	11048.15	1.00
α_{25}^{state}	-0.00	0.00	0.30	-0.64	-0.11	-0.00	0.11	0.65	10151.39	1.00
α_{26}^{state}	-0.00	0.00	0.30	-0.66	-0.12	-0.00	0.11	0.64	12545.84	1.00
α_{27}^{state}	0.00	0.00	0.29	-0.64	-0.11	0.00	0.11	0.64	14688.05	1.00
α_{28}^{state}	-0.00	0.00	0.29	-0.65	-0.11	0.00	0.11	0.63	13422.73	1.00
α_{29}^{state}	-0.00	0.00	0.30	-0.64	-0.11	-0.00	0.10	0.65	11528.96	1.00
α_{30}^{state}	-0.00	0.00	0.30	-0.67	-0.11	-0.00	0.11	0.65	13951.41	1.00
α_{31}^{state}	0.00	0.00	0.30	-0.67	-0.11	0.00	0.11	0.65	12860.85	1.00
α_{32}^{state}	-0.00	0.00	0.26	-0.54	-0.11	-0.00	0.10	0.56	6629.28	1.00
α_{33}^{state}	-0.00	0.00	0.29	-0.65	-0.11	-0.00	0.11	0.62	13174.63	1.00
α_{34}^{state}	0.00	0.00	0.29	-0.64	-0.11	-0.00	0.11	0.64	13039.06	1.00
α_{35}^{state}	0.00	0.00	0.30	-0.67	-0.11	-0.00	0.11	0.65	12938.33	1.00
α_{36}^{state}	0.00	0.00	0.29	-0.63	-0.11	0.00	0.11	0.62	14181.21	1.00
α_{37}^{state}	-0.00	0.00	0.29	-0.64	-0.11	0.00	0.10	0.64	11528.17	1.00
α_{38}^{state}	0.00	0.00	0.30	-0.64	-0.10	-0.00	0.11	0.67	13059.82	1.00
α_{39}^{state}	-0.00	0.00	0.30	-0.65	-0.11	-0.00	0.10	0.65	13000.12	1.00
α_{40}^{state}	0.01	0.00	0.30	-0.63	-0.11	0.00	0.11	0.65	12361.29	1.00
α_{41}^{state}	0.00	0.00	0.29	-0.63	-0.10	0.00	0.11	0.67	13882.77	1.00
α_{42}^{state}	-0.00	0.00	0.31	-0.70	-0.11	0.00	0.11	0.66	11855.58	1.00
α_{43}^{state}	0.00	0.00	0.31	-0.65	-0.11	0.00	0.11	0.68	13618.33	1.00
α_{44}^{state}	-0.00	0.00	0.30	-0.66	-0.11	-0.00	0.11	0.63	11607.65	1.00
α_{45}^{state}	0.00	0.00	0.31	-0.66	-0.11	0.00	0.11	0.69	9386.17	1.00
α_{46}^{state}	0.00	0.00	0.30	-0.65	-0.11	0.00	0.11	0.66	12808.94	1.00
α_{47}^{state}	0.02	0.00	0.26	-0.54	-0.09	0.01	0.12	0.62	8309.47	1.00
α_{48}^{state}	0.00	0.00	0.30	-0.64	-0.11	0.00	0.11	0.66	12433.56	1.00
α_{49}^{state}	0.00	0.00	0.30	-0.63	-0.11	0.00	0.12	0.67	13553.05	1.00
α_{50}^{state}	-0.00	0.00	0.29	-0.65	-0.11	-0.00	0.11	0.63	11518.66	1.00
α_{51}^{state}	0.08	0.01	0.27	-0.39	-0.04	0.03	0.18	0.76	1068.15	1.01
$\alpha_{r(1),1}^{region}$	0.48	0.01	0.32	-0.06	0.23	0.47	0.71	1.09	563.06	1.01
$\alpha_{r(1),2}^{region}$	0.32	0.01	0.57	-0.63	-0.04	0.21	0.63	1.67	2569.76	1.00
$\alpha_{r(2),1}^{region}$	0.46	0.01	0.31	-0.06	0.22	0.46	0.68	1.06	644.11	1.01
$\alpha_{r(2),2}^{region}$	0.41	0.01	0.53	-0.40	0.03	0.31	0.71	1.68	1578.49	1.00
$\alpha_{r(3),1}^{region}$	0.39	0.01	0.34	-0.15	0.11	0.36	0.63	1.10	642.92	1.01

$\alpha^{region}_{r(3),2}$]	-0.02	0.01	0.54	-1.14	-0.30	-0.01	0.25	1.13	8663.30	1.00
$\alpha^{region}_{r(4),1}$	0.05	0.01	0.28	-0.44	-0.14	0.01	0.23	0.67	1127.36	1.01
$\alpha^{region}_{r(4),2}$	0.27	0.01	0.56	-0.63	-0.08	0.15	0.56	1.63	2519.12	1.00
$\alpha^{region}_{r(5),1}$	0.42	0.01	0.28	-0.04	0.21	0.41	0.61	0.98	761.66	1.01
$\alpha^{region}_{r(5),2}$	0.78	0.02	0.66	-0.13	0.24	0.68	1.20	2.31	740.39	1.01
$\alpha^{region}_{r(6),1}$	0.58	0.01	0.35	-0.02	0.31	0.58	0.83	1.26	560.77	1.01
$\alpha^{region}_{r(6),2}$	0.33	0.01	0.53	-0.53	-0.02	0.23	0.62	1.58	2917.65	1.00
$\alpha^{region}_{r(7),1}$	0.49	0.01	0.31	-0.04	0.25	0.49	0.71	1.09	595.74	1.01
$\alpha^{region}_{r(7),2}$	0.30	0.01	0.50	-0.49	-0.02	0.21	0.58	1.46	2151.45	1.00
$\alpha^{region}_{r(8),1}$	0.32	0.01	0.28	-0.16	0.10	0.31	0.52	0.91	718.72	1.01
$\alpha^{region}_{r(8),2}$	0.52	0.02	0.64	-0.36	0.05	0.38	0.87	2.09	1158.01	1.00
μ	2.59	0.02	0.40	1.98	2.28	2.55	2.85	3.44	496.29	1.01
c	-0.49	0.00	0.14	-0.80	-0.58	-0.48	-0.40	-0.25	1026.26	1.01
p	0.69	0.01	0.19	0.35	0.56	0.68	0.81	1.07	1021.97	1.00

Table S8. Summary of posterior distribution from model fit. Each row is a coefficient defined in equation 2 (main text) with terminology as described in the text. Each column is a descriptor of the posterior distribution of that coefficient across all five chains, specifically: its mean, standard error, standard deviation, 2.5th, 25, 50 (median), 75, 97.5th, number of effective samples, and the $\hat{r}$ estimator of chain convergence(3).

References

1. HJT Unwin, et al., State-level tracking of COVID-19 in the United States. *Nat. Commun.* **11** (2020).
2. S Flaxman, et al., Estimating the effects of non-pharmaceutical interventions on COVID-19 in Europe. *Nature* (2020).
3. A Gelman, DB Rubin, Inference from Iterative Simulation Using Multiple Sequences. *Stat. Sci.* **7**, 457–511 (1992).
